# Clinical and imaging manifestations of intracerebral hemorrhage in brain tumors and metastatic lesions: a comprehensive overview

**DOI:** 10.1007/s11060-024-04811-2

**Published:** 2024-09-02

**Authors:** Semil Eminovic, Tobias Orth, Andrea Dell’Orco, Lukas Baumgärtner, Andrea Morotti, David Wasilewski, Melisa S. Guelen, Michael Scheel, Tobias Penzkofer, Jawed Nawabi

**Affiliations:** 1 Department of Radiology, Charité – Universitätsmedizin Berlin, Humboldt-Universität zu Berlin, Freie Universität Berlin, Berlin Institute of Health, Charitéplatz 1, 10117 Berlin, Germany; 2 Department of Neuroradiology, Charité – Universitätsmedizin Berlin, Humboldt-Universität zu Berlin, Freie Universität Berlin, Berlin Institute of Health, Berlin, Germany; 3 Department of Clinical and Experimental Sciences, Neurology Clinic, University of Brescia, Brescia, Italy; 4 Department of Neurosurgery, Charité – Universitätsmedizin Berlin, Humboldt-Universität zu Berlin, Freie Universität Berlin, Berlin Institute of Health, Berlin, Germany

**Keywords:** Brain neoplasms, Stroke, Intracerebral hemorrhage, Signs and symptoms, Diagnostic imaging

## Abstract

**Purpose:**

This observational study aims to provide a detailed clinical and imaging characterization/workup of acute intracerebral hemorrhage (ICH) due to either an underlying metastasis (mICH) or brain tumor (tICH) lesion.

**Methods:**

We conducted a retrospective, single-center study, evaluating patients presenting with occult ICH on initial CT imaging, classified as tICH or mICH on follow-up MRI imaging according to the H-Atomic classification. Demographic, clinical and radiological data were reviewed.

**Results:**

We included 116 patients (tICH: 20/116, 17.24%; mICH: 96/116, 82.76%). The most common malignancies causing ICH were lung cancer (27.59%), malignant melanoma (18.10%) and glioblastoma (10.34%). The three most common stroke-like symptoms observed were focal deficit (62/116, 53.45%), dizziness (42/116, 36.21%) and cognitive impairment (27/116, 23.28%). Highest mICH prevalence was seen in the occipital lobe (mICH: 28.13%, tICH: 0.00%; *p* = 0.004) with tICH more in the corpus callosum (tICH: 10.00%, mICH: 0.00%; *p* = 0.029). Anticoagulation therapy was only frequent in mICH patients (tICH: 0.00%, mICH: 5.21%; *p* = 0.586). Hemorrhage (tICH: 12682 mm^3^, mICH: 5708 mm^3^, *p* = 0.020) and edema volumes (tICH: 49389 mm^3^, mICH: 20972 mm^3^, *p* = 0.035) were significantly larger within tICH patients.

**Conclusion:**

More than half of the patients with neoplastic ICH exhibited stroke-like symptoms. Lung cancer was most common in mICH, glioblastoma in tICH. While clinical presentations were similar, significant differences in tumor location and treatments were discernible.

**Supplementary Information:**

The online version contains supplementary material available at 10.1007/s11060-024-04811-2.

## Introduction

Central nervous system (CNS) tumors and metastases, encompassing those of the brain, are among the most lethal cancers, contributing significantly to morbidity and mortality [1–3]. A subgroup of significant clinical relevance, termed ‘neoplastic intracerebral hemorrhage’, arises from rhexis bleeding of fragile neo-angiogenetic tumor vessels within neoplastic brain tumors or metastases. This phenomenon has garnered increasing attention in recent clinical studies due to its relatively obscure nature and its association with elevated mortality rates [4–6]. Distinguishing neoplastic intracerebral hemorrhages from other etiologies, such as hypertensive parenchymal bleedings, presents a clinical challenge [4, 7–13]. This difficulty may lead to delayed diagnostic follow-up and the postponement of critical, time-sensitive treatment options, culminating in generally poor patient outcomes [4]. Efforts to distinguish neoplastic intracerebral hemorrhages from their non-neoplastic counterparts have been ongoing, with quantitative imaging strategies yielding encouraging outcomes [14–16]. Despite these advancements, a comprehensive understanding of the precise incidence and clinical features of these hemorrhages is yet to be fully realized. Enhanced delineation of their incidence and clinical presentation is imperative for improving diagnostic accuracy and subsequent patient care.

We aim to provide a detailed clinical and imaging characterization of acute intracerebral hemorrhage (ICH) due to a neoplastic brain lesion. Within the scope of this we hypothesize that patients with neoplastic ICH will present with clinical symptoms similar to those observed in patients with other cerebrovascular stroke events. Furthermore, we predict that there will be distinct clinical and radiographic characteristics differentiating ICH associated with primary brain tumors (tICH) from ICH associated with metastatic lesions (mICH), which could be instrumental in establishing an accurate and timely diagnosis. For this purpose, we analyzed characteristics of intracerebral hemorrhage in brain tumors and metastatic lesions, across demographics, symptoms, imaging findings, treatment, and outcomes.

## Methods

This single center retrospective study was approved by the ethics committee (Charité Berlin, Germany [protocol number EA1/035/20]) and written informed consent was waived by the institutional review boards. All study protocols and procedures were conducted in accordance with the Declaration of Helsinki. Patient consent was not needed due to the retrospective nature of the study.

### Study population

In the current analysis, we focused on a subset of patients derived from a previously published cohort at Charité University Hospital in Berlin, Germany, from January 2016 to May 2020 [16]. The inclusion process for this subgroup followed the protocols detailed in our earlier study, employing the H-ATOMIC Classification system to categorize the etiology of occult, acute ICH [17]. The refined inclusion process for this patient subset was restricted to parenchymal ICH cases with confirmed neoplastic origins, excluding hemorrhages caused by hypertension, cerebral amyloid angiopathy (CAA), coagulopathies, vascular malformations, hemorrhagic transformation post-ischemic stroke, trauma, cryptogenic factors, unconfirmed diagnoses, or any cases with prior cranial surgery. The present analysis extends our initial findings by incorporating a detailed examination of the specific neoplastic pathologies of ICH, as verified by final medical reports, thereby providing a precise classification of ICH causes within this subgroup of primary brain tumors and secondary metastases.

### Clinical characteristics

Clinical data for this specific subgroup were extracted from medical records, capturing demographic information (average age, sex, blood pressure), Glasgow Coma Scale (GCS) at admission and modified Rankin Scale (mRS) at last medical evaluation or at discharge. Additionally, vascular risk factors (hypertension, diabetes mellitus), medication already taken before ICH (oral coagulation, antiplatelet therapy (acetylsalicylic acid (asa) or clopidogrel) and therapies such as craniotomy, chemo- and/or immunotherapy, radiotherapy, external ventricular drainage placement, steroid therapy prior and after bleeding event were obtained from patients’ clinical records.

We also collected clinical findings for every patient included as follows: focal deficit, seizure, coma, confusion/cognitive impairment, headache, dizziness/gait impairment, non-specific/systemic symptoms like fever or weight loss as these are no common symptoms related to ICH [18, 19], but still were found at least in a minor set of patients in our cohort. Furthermore, we reviewed whether these symptoms have occurred for the first time. We have also determined the respective primary tumors for each patient and are looking at metastases of lung cancer, malignant melanoma, breast cancer, prostate cancer, pancreatic cancer, colorectal cancer, renal cell cancer, cancer of unknown primary, a few other primary tumors as well as glioblastoma, astrocytoma, oligodendroglioma, meningioma and lymphoma as primary tumors of the CNS. MRS was dichotomized into 0–3 and 4–6 because the difference between these two categories is medically relevant. Patients with mRS between 0 and 3 maintain some degree of independence in the activities of daily living, while patients with scores > 3 need complete assistance [20].

### Radiological characteristics

Informed by the imaging dataset of our previously detailed cohort [16], which was subjected to thorough image quality assessment and volumetric quantification of ICH and perihematomal edema (PHE) at baseline, our current study was expanded to include the evaluation of secondary intraventricular hemorrhage (IVH) and the detailed localization of hemorrhagic sites. The criteria for classifying hemorrhage locations were systematically derived, incorporating the identification of multiple hemorrhages, the determination of lateralization (left, right or bilateral) and the involvement of distinct cerebral regions, including the frontal, temporal, parietal and occipital lobes, as well as the corpus callosum, basal ganglia/thalamus, brainstem, cerebellum, and sella [21]. All bleeding events included in our study are strictly intra-tumoral. However, it is possible that the hemorrhage on initial CT may have “masked” the underlying neoplastic lesion, which only became evident on the follow-up MRI, but was not apparent on the initial CT scan.

### Statistical analysis

For numeric data, mean and standard deviations (SD) were used for normally distributed data and median and interquartile range otherwise. Distributions for numeric data were compared using an, two-tailed, two sampled (tumor and metastases group) t-test. Normality assumptions were confirmed using a Shapiro-Wilk-Test or assumed based of central limit theorem. If these assumptions were violated, the Mann-Whitney U test was applied instead. Categorical variables like the occurrence of a specific symptom were compared using the χ^2^ test or the fisher test with one degree of freedom where the χ^2^ test assumption were not met [22]. P-values less than 0.05 were considered significant. Analyses were performed using the statistical software package R (programming language, version 4.3.1 (2023-06-16)), www.R-project.orgInt. Graphs and diagrams were created with tidyverse [23] (version 2.0.0), a collection of packages within R (core packages dplyr 1.1.2, readr 2.1.4, forcats 1.0.0, stringr 1.5.0, ggplot2 3.4.2, tibble 3.2.1, lubridate 1.9.2, tidyr 1.3.0, purr 1.0.2) of which in particular ggplot2 [24] (version 3.4.2) and dplyr [25] (version 1.1.2) was used. Figure 4 was created with biorender (https://app.biorender.com).

## Results

**Fig. 1:**
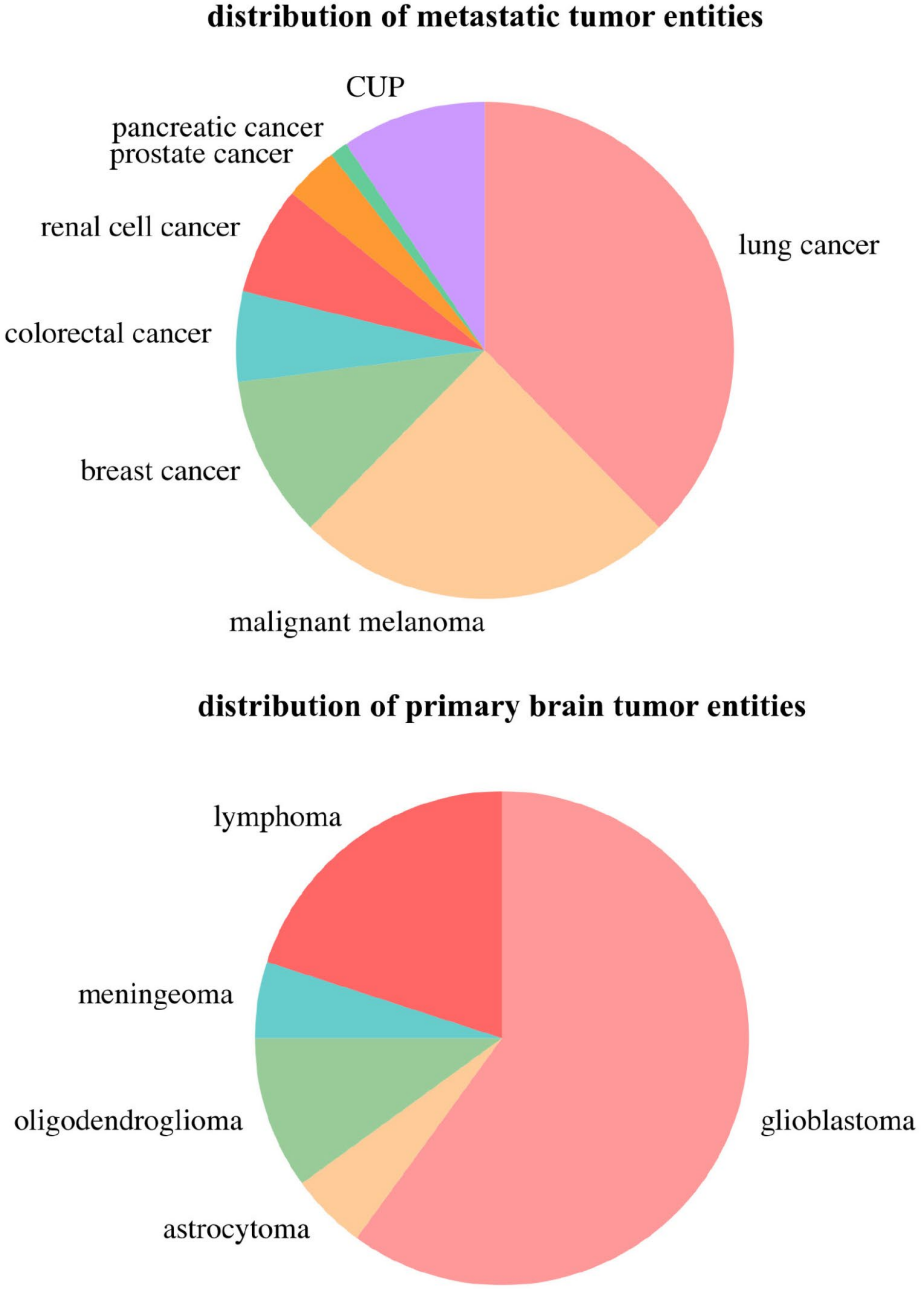
Pie charts for the distribution of neoplastic hemorrhage entities. Each segment represents the proportion of cases for a specific type of tumor or metastasis associated with hemorrhage. **Top Chart** Distribution of metastatic entities, excluding primary brain tumors. Lung cancer, malignant melanoma and breast cancer are among the most prevalent types depicted. **Bottom Chart** Distribution of primary brain tumor entities is displayed in this pie chart

In this study, we included 116 patients, of whom 20 (17.24%) had ICH from primary brain tumor bleeding (tICH), the other 96 (82.76%) had ICH from metastatic bleeding (mICH). Table 1 shows the general characteristics of both the whole study population or the individual groups (tICH vs. mICH). The four most common tumor entities that led to ICH were lung cancer (32/116, 27.59%), malignant melanoma (21/116, 18.10%), glioblastoma (12/116, 10.34%) and breast cancer (9/116, 7.76%) as illustrated in Fig. 1.

**Table 1:** Comparative analysis of study populations: intracerebral hemorrhage from primary tumor bleeding versus metastatic bleeding

	All (*N* = 116)	Tumor (N1 = 20)	Metastasis (N2 = 96)	T-value t-test	*P*-value MWU-test	*P*-value χ^2^ test	*P*-value fisher test
**Demographics**							
Average age (Median, IQR)	62.5 (51.00–77.00)	60.5 (46.25–73.25)	62.5 (53.00–77.00)		**0.269**		
Sex, n (ref: female)	64 (55.17%)	13 (65.00%)	51 (53.13%)			**0.469**	
GCS at admission, median (IQR)	15 (15–15)	15 (15–15)	15 (15–15)		**0.410**		
Blood pressure, mean (SD)	140.86 (25.08)	132 (24.44)	142.75 (24.97)	**0.126**			
Diabetes, n (%)	7 (6.03%)	0 (0.00%)	7 (7.29%)				**0.602**
Hypertension, n (%)	39 (33.62%)	6 (30.00%)	33 (34.38%)			**0.907**	
Oral anticoagulation, n (%)	5 (4.31%)	0 (0.00%)	5 (5.21%)				**0.586**
Antiplatelet therapy (asa, clopidogrel), n (%)	12 (10.34%)	0 (0.00%)	12 (12.50%)				**0.124**

Clinical findings	All (*N* = 116)	Tumor (N1 = 20)	Metastasis (N2 = 96)	T-value t-test	P-value MWU-test	P-value χ^2^test	P-value fisher-test
Focal deficit, n (%)	62 (53.45%)	12 (60.00%)	50 (52.08%)			**0.690**	
Seizure, n (%)	23 (19.83%)	6 (30.00%)	17 (17.71%)			**0.344**	
Coma, n (%)	1 (0.86%)	1 (5.00%)	0 (0.00%)				**0.384**
Confusion/cognitive impairment, n (%)	27 (23.28%)	8 (40.00%)	19 (19.79%)			**0.098**	
Headache, n (%)	24 (20.69%)	3 (15.00%)	21 (21.88%)				**0.762**
Dizziness/gait impairment, n (%)	42 (36.21%)	7 (35.00%)	35 (36.46%)			**1.000**	
Non-specific/systemic symptoms, n (%)	14 (12.07%)	2 (10.00%)	12 (12.50%)				**0.305**
First clinical manifestation, n (%)	24 (20.69%)	3 (15.00%)	21 (21.88%)				**0.830**

Radiological findings	All (*N* = 116)	Tumor (N1 = 20)	Metastasis (N2 = 96)	T-value t-test	P-value MWU-test	P-value χ^2^test	P-value fisher-test
Baseline ICH volume [mm^3^], median (IQR)	6854.39 (2316.33-19788.10)	12682.24 (5180.11-29393.94)	5707.88 (2072.57-16952.21)		0.020 (*)		
Baseline PHE volume [mm^3^], median (IQR)	23008.57 (13338.98-59241.96)	49388.53 (22267.62-61315.77)	20972.12 (10169.56-58606.73)		0.035 (*)		
Baseline IVH presence, n (%)	7 (6.03%)	1 (5.00%)	6 (6.25%)				**1.000**

Bleeding location	All (*N* = 116)	Tumor (N1 = 20)	Metastasis (N2 = 96)	T-value t-test	P-value MWU-test	P-value χ^2^test	P-value fisher-test
Multiple bleedings, n (%)	36 (31.03%)	4 (20.00%)	32 (33.33%)				**0.198**
Side, n (%) Left, n (%) Right, n (%) Bilateral, n (%)							
	40 (34.48%)	7 (35.00%)	33 (34.38%)			**1.000**	
	39 (33.62%)	10 (50.00%)	29 (30.21%)			**0.149**	
	37 (31.90%)	3 (15.00%)	34 (35.42%)			**0.129**	
Frontal lobe, n (%)	62 (53.45%)	13 (65.00%)	49 (51.04%)			**0.372**	
Temporal lobe, n (%)	30 (25.86%)	8 (40.00%)	22 (22.92%)			**0.191**	
Parietal lobe, n (%)	47 (40.52%)	5 (25.00%)	42 (43.75%)			**0.192**	
Occipital lobe, n (%)	27 (23.28%)	0 (0.00%)	27 (28.13%)				0.004 (**)
Corpus Callosum, n (%)	2 (1.72%)	2 (10.00%)	0 (0.00%)				0.029 (*)
Basal Ganglia/Thalamus, n (%)	15 (12.93%)	5 (25.00%)	10 (10.42%)			**0.161**	
Brainstem, n (%)	4 (3.45%)	0 (0.00%)	4 (4.17%)				**1.000**
Cerebellum, n (%)	26 (22.41%)	0 (0.00%)	26 (27.08%)				0.006 (**)
Sella, n (%)	0 (0.00%)	0 (0.00%)	0 (0.00%)				**1.000**
Insular region, n (%)	4 (3.45%)	1 (5.00%)	3 (3.13%)				**1.000**

Therapy	All (*N* = 116)	Tumor (N1 = 20)	Metastasis (N2 = 96)	P-value t-test		P-value χ^2^test	P-value fisher-test
Craniotomy, n (%)	57 (49.14%)	14 (70.00%)	43 (44.79%)			**0.071**	
Chemo-/Immunotherapy, n (%)	21 (18.10%)	8 (40.00%)	13 (13.54%)			0.013 (*)	
Radiotherapy, n (%)	41 (35.34%)	6 (30.00%)	35 (36.46%)			**0.770**	
EVD placement, n (%)	3 (2.59%)	0 (0.00%)	3 (3.13%)				**1.000**
Steroidtherapy prior bleeding event, n (%)	13 (11.21%)	2 (10.00%)	11 (11.46%)				**1.000**
Steroidtherapy after bleeding event, n (%)	78 (67.24%)	10 (50.00%)	68 (70.83%)			0.020 (*)	

Clinical outcome	All (*N* = 116)	Tumor (N1 = 20)	Metastasis (N2 = 96)	P-value t-test	P-value MWU-test	P-value χ^2^test	P-value fisher-test
mRS (dichotomized [0="mRS < = 3”, 1="mRS > 3”], n (%)	11 (9.48%)	2 (10.00%)	9 (9.38%)				**1.000**
**Etiology [%]**							
***Brain Metastasis***	*N* = 96		[%] metastasis	[%] all			
Lung cancer, n	32	-	33.33%	27.59%			
Malignant melanoma, n	21	-	21.88%	18.10%			
Breast cancer, n	9	-	9.38%	7.76%			
Prostate cancer, n	3	-	3.13%	2.59%			
Pancreatic cancer, n	1	-	1.04%	0.86%			
Colorectal cancer, n	5	-	5.21%	4.31%			
Renal cell cancer, n	6	-	6.25%	5.17%			
Cancer of unknown primary, n	8	-	8.33%	6.90%			
Other, n	11	-	11.46%	9.48%			
***Primary brain tumor***	*N* = 20	[%] primary brain tumor					
Glioblastoma, n	12	60%	-	10.34%			
Astrocytoma, n	1	5%	-	0.86%			
Oligodendroglioma, n	2	10%	-	1.72%			
Meningeom, n	1	5%	-	0.86%			
Lymphom, n	4	20%	-	3.45%			

*: significant (*p* < 0.05); **: *p* < 0.01; ns: *p* > 0.05

*Legend* SD, standard deviation; MWU, Mann-Whitney-U-Test; χ^2^, chi square; IQR, interquartile range; GCS, Glasgow Coma Scale; ASA, acetylsalicylic acid; ICH, intracerebral hemorrhage; mRS, modified Rankin Scale; PHE, perihematomal edema; EVD, external ventricular drain

### Demographics

Oral anticoagulation (tICH: 0/20 [0.00%], mICH: 5/96 [5.21%]; *p* = 0.586) and antiplatelet therapy (tICH: 0/20 [0.00%], mICH: 12/96 [12.50%]; *p* = 0.124) were both only frequent in the mICH cohort.

### Clinical findings

**Fig. 2:**
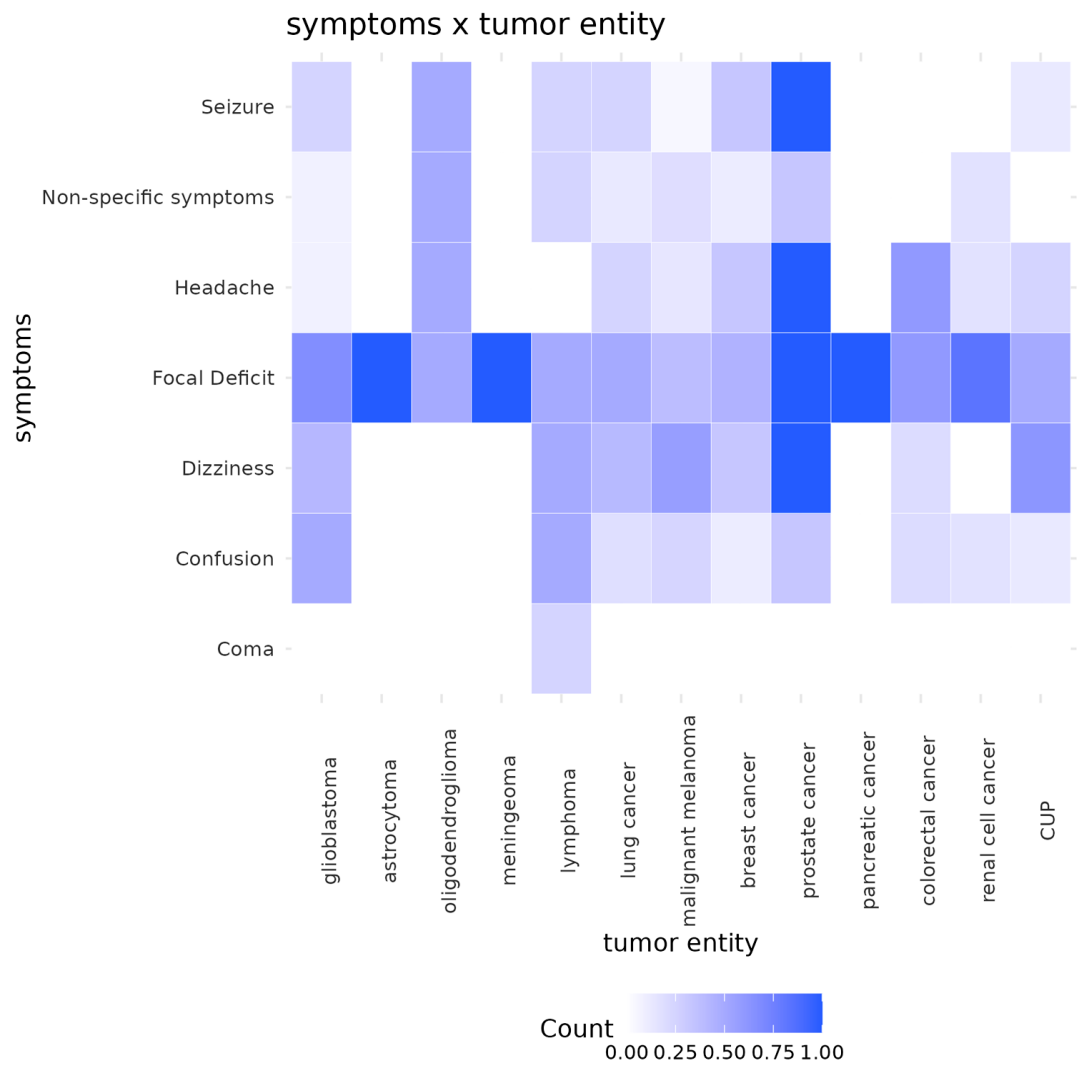
Heatmap of symptom distribution per tumor entity. Deeper shades of purple indicating a higher incidence of the respective symptom among the tumor entities, i.e. only one patient with a bleeding secondary to brain metastases had underlying pancreatic cancer, as there was only one patient representing this category, and they exhibited a focal deficit, the symptom frequency for this tumor entity is marked as 100%, resulting in a particularly dark field on the heatmap

Hemorrhagic stroke can vary widely in symptomatic appearance, but in summary it can present with (i) general symptoms such as decreased level of consciousness, nausea, vomiting, headache or seizures due to increased intracranial pressure; and (ii) with focal neurologic impairments according to the ICH location [26, 27]. Our patient cohort showed many of those classical hemorrhagic stroke symptoms as the three most common displayed were focal deficit (62/116, 53.45%), dizziness (42/116, 36.21%) and cognitive impairment (27/116, 23.28%). No substantial differences were observed in the overall clinical symptoms between the two groups. Focal deficit emerged as the predominant clinical symptom within both groups (tICH: 12/20 [60.00%], mICH: 50/96 [52.08%]; *p* = 0.690). The presence of focal deficit was consistent across all tumor types as illustrated in Fig. 2. We recorded focal deficit as a set of symptoms in which causation can be localized to an anatomic site in the central nervous system [28], e.g. paralysis of a muscle or a group of muscles. Notably, for certain tumor entities, including astrocytoma, meningioma and prostate cancer with CNS involvement, focal deficit was even the only clinical manifestation. Seizure was reported in 30.00% in the tICH group, in contrast to 17.71% in the mICH group (*p* = 0.344). Cognitive impairment was found in 8 (40.00%) of the primary tumor patients, compared to 19 (19.79%) in metastatic patients (*p* = 0.098). Dizziness was observed relatively frequently in both groups (tICH: 7/20 [35.00%], mICH: 35/96 [36.46%]; *p* = 1.000). Figure 2 provides a nuanced visualization through a heatmap, delineating the distribution of clinical symptoms across different tumor entities.

### Radiological findings

The median baseline ICH volume (tICH: 12682 [5180–29394] mm^3^, mICH: 5708 [2073–16952] mm^3^; *p* = 0.020) and the baseline perihematomal edema volume (tICH: 49389 [22268–61316] mm^3^, mICH: 20972 [10170–58607] mm^3^; *p* = 0.035) were significantly larger in the primary tumor group than in the metastases group. In the supplementary material, there are detailed case-by-case images for different tumor entities (non-contrast CT, MRI post-contrast T1w, MRI T2w).

### Bleeding locations

**Fig. 3:**
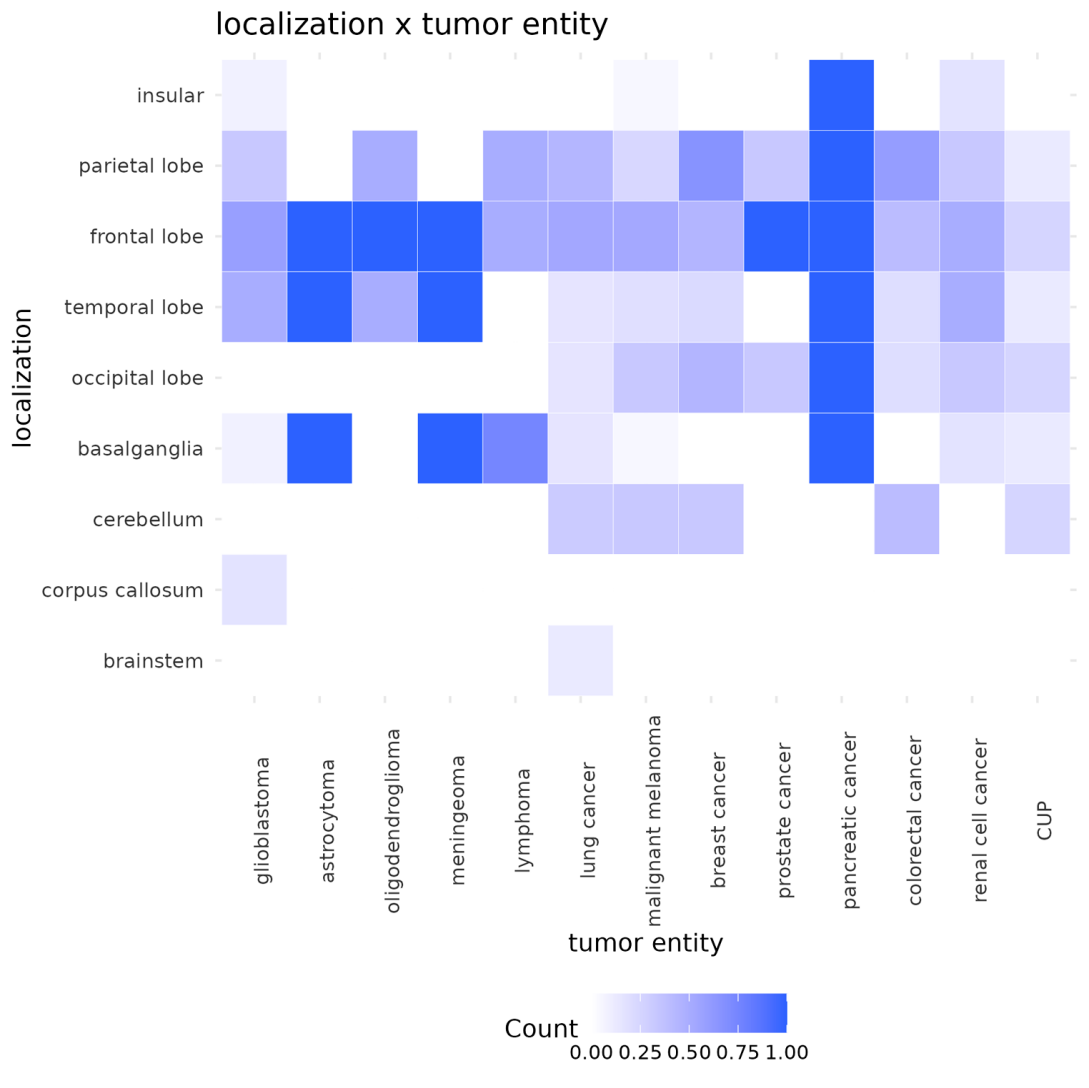
Heatmap of localization distribution per tumor entity. Deeper shades of purple indicating a higher incidence of the respective tumor entity within certain brain regions. For instance, the most prevalent site of hemorrhage in patients with glioblastoma was the frontal lobe

**Fig. 4:**
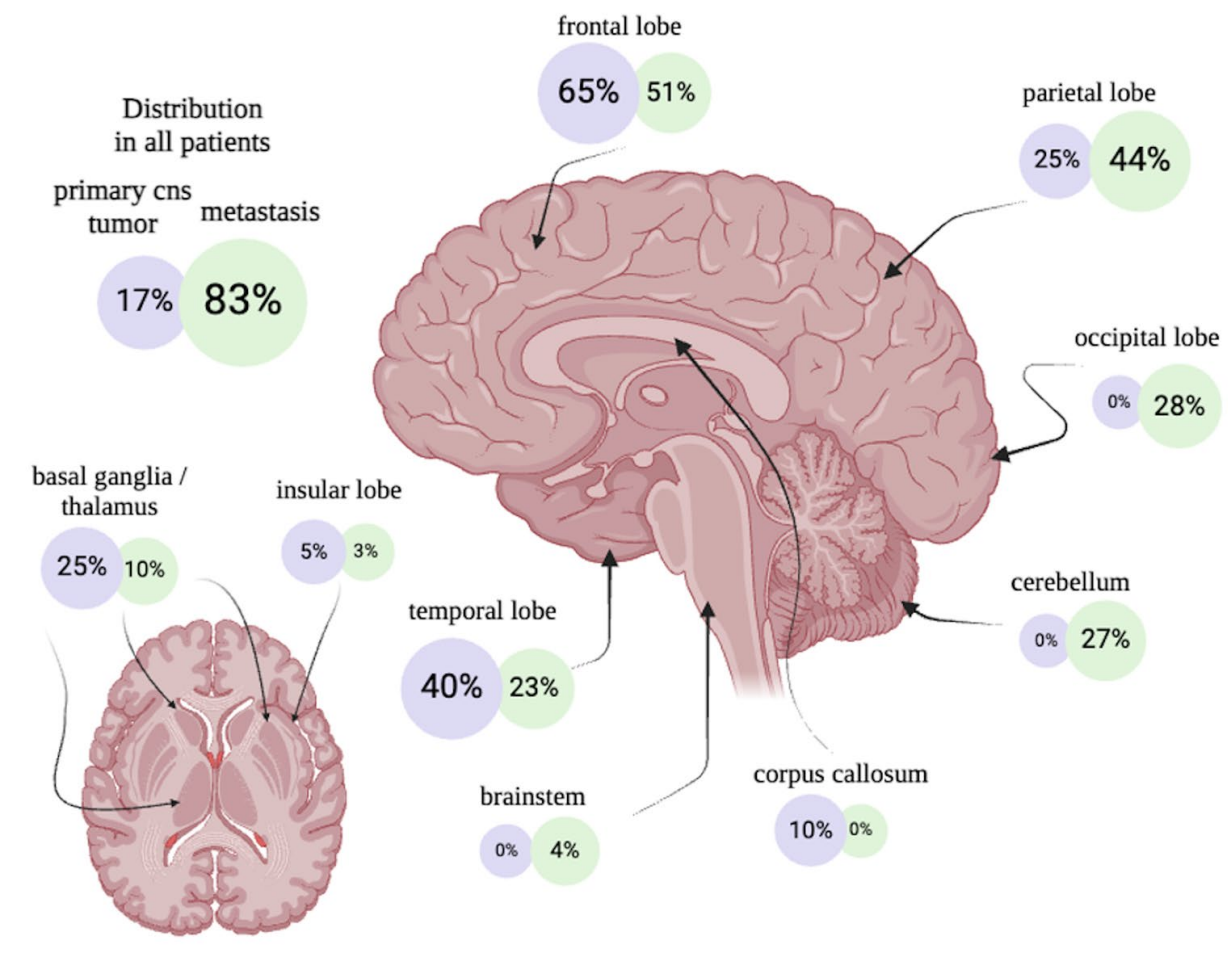
Illustration of the localization frequency of hemorrhagic primary cns tumors vs. metastatic lesions, e.g. 65% of all hemorrhaged primary cns tumors were localized in the frontal lobe in contrast to 51% of hemorrhaged brain metastases. It should be noted that the cumulative percentages may exceed 100% for each category, as individual patients could present with multiple hemorrhagic sites

Notably, only metastatic lesions exhibited hemorrhages in the occipital lobe (mICH: 27/96 [28.13%], ICH: 0/20 [0.00%]; *p* = 0.004) and the cerebellum (mICH: 26/96 [27.08%], tICH: 0/20 [0.00%]; *p* = 0.006). Remarkably, in those specific brain regions, no instances of hemorrhage were observed in association with primary tumors. In addition, no tICH was found in the brainstem, although this occurrence was not statistically distinct from that observed in mICH (tICH: 0/20 [0.00%], mICH: 4/96 [4.17%]; *p* = 1.000), those four mICH patients had their metastasis originating from a lung carcinoma. Conversely, primary brain tumors were found to have a significantly greater frequency of hemorrhages within the corpus callosum (tICH: 2/20 [10.00%], mICH: 0/96 [0.00%]; *p* = 0.0285). Corpus callosum was affected only in two cases of glioblastomas. Figure 3 offers a comprehensive heatmap detailing the distribution of hemorrhage localizations across different tumor entities. Interestingly, bleedings within the frontal lobe were a common feature with all tumor entities. 65.00% of all tICH were localized in the frontal lobe in contrast to 51.04% of mICH without a significant difference between the two groups (*p* = 0.372). Figure 4 shows a detailed illustration of the localization frequency differentiating between tICH and mICH.

### Therapy

Patients with hemorrhagic primary brain tumors demonstrated a significantly higher frequency of undergoing chemotherapy or immunotherapy (tICH: 8/20 [40.00%], mICH: 13/96 [13.54%]; *p* = 0.013). In contrast, patients with mICH exhibited a significantly increased likelihood of being administered steroid therapy post-ICH (mICH: 68/96 [70.83%], tICH: 10/20 [50.00%]; *p* = 0.020). Furthermore, craniotomy was performed more frequently within the tICH-group (tICH: 14/20 [70.00%], mICH: 43/96 [44.79%]; *p* = 0.071), albeit not to a statistically significant degree.

## Discussion

In this study, we investigated the clinical and imaging characteristics of neoplastic ICH related to brain tumors and metastatic lesions. Our primary objective was to provide a comprehensive clinical and imaging characterization of acute ICH due to either an underlying metastasis or brain tumor lesion. In the course of this as a secondary objective we compared those cohorts to elucidate any distinct pathological and clinical nuances between brain tumor-related ICH and metastatic ICH.

### Clinical characteristics

Our findings showed that stroke-like presentation was very common within tumor-related ICH as many patients presented with classical hemorrhagic stroke symptoms such as focal neurological deficits and general symptoms like cognitive impairment, seizures and dizziness. Our findings underscore the clinical significance, aligning with the growing consensus in the literature that ICH may present as the initial clinical manifestation in patients harboring an occult tumor. These tumors, which remain undetected until they manifest with complications like hemorrhage, pose a diagnostic challenge.

### Etiology characteristics

As shown in Fig. 1, the distribution of tumor entities that led to ICH in this study also aligned well with the existing literature [29, 30]: The most prevalent primary malignancies associated with ICH included lung cancer, which was the most common, followed by melanoma, glioma, breast cancer, leukemia, and renal carcinoma. Their frequent association with ICH could be partly attributed to their high overall incidence and significant role in brain metastases. Although renal cell carcinoma, testicular, hepatocellular, and thyroid cancers are known for their tendency to bleed, only renal cell carcinoma was commonly represented in this study [31]. Despite fewer cases of tICH, glioblastomas were notably prevalent within this group. This is consistent with glioblastoma’s status as the most common type of brain tumor, distinguished by its marked angiogenesis and its aggressive, invasive, and destructive profile [12, 32–35]. Only patients in the mICH cohort received oral anticoagulation and antiplatelet therapy with those therapies being a potential risk factor for ICH. That being said, in their meta-analysis Giustozzi et al. [36] showed that anticoagulant therapy is associated with an increase in ICH in patients with primary brain cancer, but not in those with brain metastases. Further studies suggested that antiplatelet therapy was not associated with increased risk of ICH in patients with metastatic brain tumors [37] or primary brain tumors [38].

### Anatomical characteristics

The prominence of tICH within the corpus callosum may be due to the frequent occurrence of glioblastomas in this patient cohort, as they are known to infiltrate the corpus callosum [39], which is generally relatively resistant to infiltration [40], particularly when compared to the rarity of corpus callosum infiltration by brain metastases [41]. Furthermore, the anatomical distribution of tICH and mICH in our study correlates with the established literature. We found that 65% of tICH cases occurred in the frontal lobe, mirroring the expected distribution pattern of these tumors—most frequently in the frontal lobe, followed by the temporal, parietal, and then occipital lobes [42]. Furthermore, while tumors in the cerebellum constitute the majority of CNS tumors in children, they are uncommon in adults [43]. Similarly, the distribution of mICH in our study confirmed the patterns reported in the literature, which suggest a more evenly dispersed distribution across the brain, with a notable involvement of the occipital lobe and cerebellum in comparison to primary CNS tumors [44].

### Imaging characteristics

As for the imaging characteristics, our study evaluated the utility of ICH and PHE in the subgroup-specific differentiation of mICH and tICH. Our findings complemented previous findings reported by Nawabi et al., which highlighted the potential of these quantitatively measured parameters to distinguish between neoplastic and non-neoplastic causes of ICH [14–16]. IVH occurs in 30–50% of primary ICH cases [45–48], in contrast to only 5–6% within our study cohort, indicating that IVH is a relatively rare occurrence and is typically linked to ICH in the basal ganglia due to hypertensive hemorrhage [48]. Nevertheless, the presence of IVH does not exclude a neoplastic etiology. We acknowledge that no clear link can be established between the observed clinical symptoms and imaging findings, as those differences in volumes and also locations may have contributed to the different set of clinical symptoms observed which need further investigation.

### Treatment characteristics

The preference for craniotomy in tICH treatment reflects the rarity of primary brain tumors presenting at multiple sites, with multifocal glioblastoma incidences ranging from 0.5 to 35% [49–52]. This contrasts with brain metastases, where 30–50% of patients may have multiple lesions [53, 54]. Additionally, the higher incidence of chemo-/immunotherapy in tICH patients in this study suggests a potential association with an increased risk of hemorrhagic transformation in primary brain tumors.

## Limitations

As this study aims to provide a detailed clinical and imaging characterization of acute neoplastic ICH we did not include a comparative non-neoplastic ICH cohort with therefore limited ability to directly compare those two groups. Based on the infrastructural connection to the large skin tumor center Charité there could be a selection bias due to a non-representative high number of included melanoma patients. Given the high mortality associated with ICH, there is a potential for survivorship bias, as patients with more severe or lethal findings, such as larger hematomas, may be underrepresented. Additionally, distinguishing a hemorrhagic neoplasm from a non-neoplastic ICH on initial CT imaging can be challenging, potentially leading to the exclusion of some patients who were mistakenly misclassified, and thus not included in the study if follow-up MRI was not performed or available. Therefore, patients who did not undergo the full range of imaging or had incomplete documentation may have been excluded, possibly leading to a non-representative sample. Our study was conducted in a single tertiary care hospital, which may have led to an overrepresentation of patients with more severe or advanced conditions due to referral patterns. This may skew the findings towards more complex or critical cases, potentially underrepresenting patients with milder forms of neoplastic ICH. Given that patients with coagulopathies, and trauma were excluded, there is a potential selection bias that limits the generalizability to cases where multifactorial causes are involved. Additionally, the exclusion of patients with prior cranial surgery may overlook those whose neoplasms presented initially with ICH post-surgery, thereby missing this important subset. The study timeframe (January 2016 to May 2020) could introduce temporal selection bias. Advancements in imaging technology and treatment protocols during this period might have influenced both the detection rates and management of neoplastic ICH, which may not fully reflect current practices. Moreover, we considered a relatively small sample size of tICH compared to the mICH cohort, which may impact the generalizability of the results and limit their ability to detect subtle differences or associations. Further studies may validate our findings on a larger cohort. Finally, the clinical significance of hemorrhagic events within our cohort remains indeterminate, necessitating additional research to corroborate their relevance in a clinical context.

## Conclusion

ICH may serve as a primary indication of an underlying brain tumor, highlighting the critical need for precise diagnostic evaluation and rapid choice of appropriate subsequent imaging. This study focused on neoplastic ICH with the purpose to provide a comprehensive clinical and imaging characterization. In the course of this we also compared primary brain tumors and metastatic lesions to outline potential differences. Our results suggest that stroke-like symptoms are relatively common; nevertheless, these may concomitantly present with non-specific manifestations including cognitive dysfunction and dizziness. Such clinical presentations may be instrumental in distinguishing neoplastic intracerebral hemorrhage from non-neoplastic etiologies. Although symptomatic presentations did not significantly vary between mICH and tICH, distinct differences in tumor localization and radiological metrics were observed. Hemorrhage and edema volumes were significantly larger within tICH patients which may have contributed to differences observed in the clinical symptoms, and particularly IVH, traditionally not associated with neoplastic ICH, presented as an imaging characteristic, suggesting it merits consideration in diagnostic deliberations. Such distinctions underscore the necessity to refine diagnostic protocols to enhance the identification of neoplastic ICH.

## Electronic supplementary material

Below is the link to the electronic supplementary material.

Supplementary Material 1

## Data Availability

The datasets generated during and/or analysed during the current study are available from the corresponding author on reasonable request.
