# Supplementary material for: Clinical and imaging manifestations of intracerebral hemorrhage in brain tumors and metastatic lesions: a comprehensive overview
Source: J Neurooncol. 2024 Sep 2;170(3):567–78. doi: 10.1007/s11060-024-04811-2 (PMC11614960; doi:10.1007/s11060-024-04811-2)
Supplement: Supplementary file 1 — Supplementary Material 1 [file 11060_2024_4811_MOESM1_ESM.docx]

Clinical and Imaging Manifestations of Intracerebral Hemorrhage in Brain Tumors and Metastatic Lesions: A comprehensive Overview

Semil Eminovic MD B.Sc.^1^, Tobias Orth^1^, Andrea Dell’Orco M.Sc.^2^, Lukas Baumgärtner Dipl Ing.^1^, Andrea Morotti MD^3^, David Wasilewski MD^4^, Melisa S. Guelen^2^, Michael Scheel MD^2^, Tobias Penzkofer MD^1*^, Jawed Nawabi MD MHBA^2*^

^1^ Department of Radiology, Charité – Universitätsmedizin Berlin, Humboldt-Universität zu Berlin, Freie Universität Berlin, Berlin Institute of Health, Berlin, Germany.

^2^ Department of Neuroradiology, Charité – Universitätsmedizin Berlin, Humboldt-Universität zu Berlin, Freie Universität Berlin, Berlin Institute of Health, Berlin, Germany.

^3^ Department of clinical and experimental sciences, Neurology Clinic, University of Brescia, Brescia, Italy.

^4^ Department of Neurosurgery, Charité – Universitätsmedizin Berlin, Humboldt-Universität zu Berlin, Freie Universität Berlin, Berlin Institute of Health, Berlin, Germany.

* = these authors contributed equally

**Corresponding Author**

Semil Eminovic, MD B.Sc.

Department of Radiology

Universitätsmedizin Berlin

Charitéplatz 1

10117 Berlin. Germany

Phone: +49-1792498327

Email: [semil.eminovic@charite.de](mailto:semil.eminovic@charite.de)

ORCID: 0009-0001-1871-2976

**Supplementary material**

**Supplementary Figure 1:** Representative cases of neoplastic intracerebral hemorrhage (green dot) linked to lung cancer, malignant melanoma, and breast cancer are depicted. The left panel shows non-contrast Computed Tomography (CT), the middle panel displays post-contrast T1-weighted (T1w) imaging, and the right panel features T2-weighted (T2w) imaging.

**Supplementary Figure 2:** Representative cases of neoplastic intracerebral hemorrhage (green dot) linked to glioblastoma, oligodendroglioma, and cns lymphoma are depicted. The left panel shows non-contrast Computed Tomography (CT), the middle panel displays post-contrast T1-weighted (T1w) imaging, and the right panel features T2-weighted (T2w) imaging.

**Supplementary Figure 3:** Representative cases of neoplastic intracerebral hemorrhage linked to adrenal cancer, liposarcoma, and cholangiocarcinoma are depicted. The left panel shows non-contrast Computed Tomography (CT), the middle panel displays post-contrast T1-weighted (T1w) imaging, and the right panel features T2-weighted (T2w) imaging.

**Supplementary Figure 4:** Representative cases of neoplastic intracerebral hemorrhage (green dot) linked to renal cell cancer, rectal cancer, and endometrial cancer are depicted. The left panel shows non-contrast Computed Tomography (CT), the middle panel displays post-contrast T1-weighted (T1w) imaging, and the right panel features T2-weighted (T2w) imaging.

**Supplementary Figure 5:** Representative cases of neoplastic intracerebral hemorrhage (green dot) linked to meningioma, astrocytoma, and glioblastoma are depicted. The left panel shows non-contrast Computed Tomography (CT), the middle panel displays post-contrast T1-weighted (T1w) imaging, and the right panel features T2-weighted (T2w) imaging.


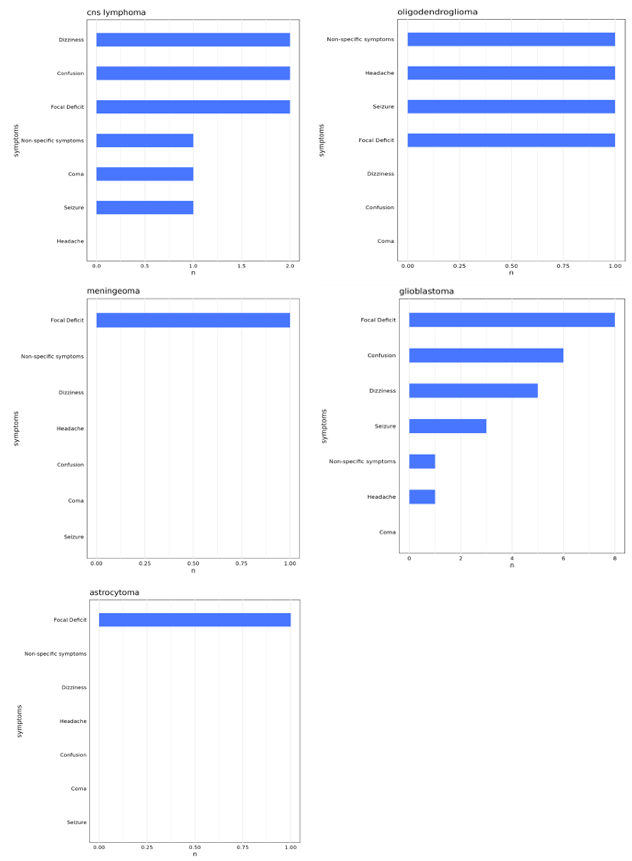


**Supplementary Figure 6:** Symptom distribution by hemorrhaged primary brain neoplastic entities. Bar charts represent the counts of clinical symptoms for patients with underlying neoplastic ICH. Each bar indicates the number of patients (n) presenting with the listed symptom.


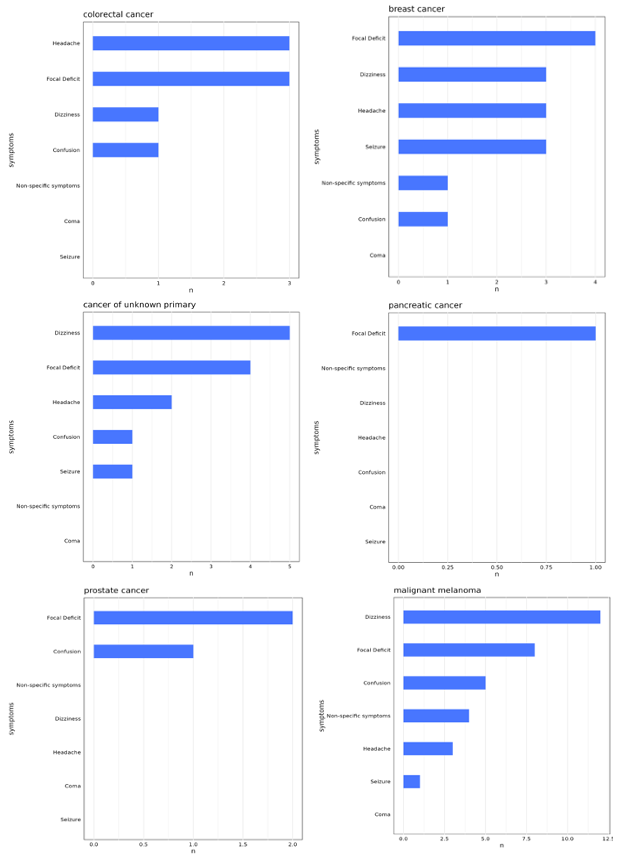


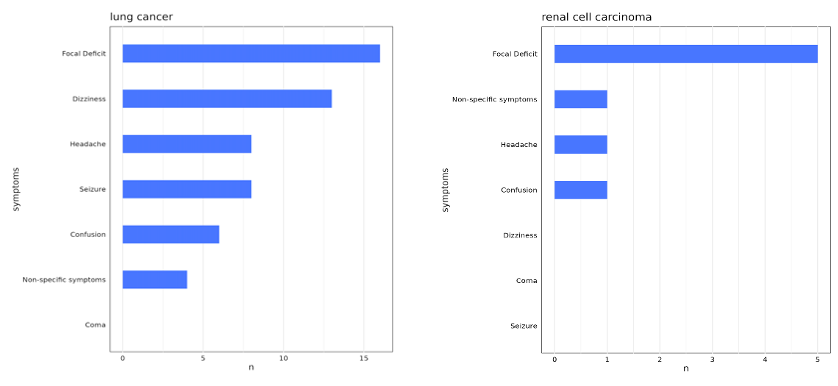


**Supplementary Figure 7:** Symptom distribution by hemorrhaged neoplastic entities without primary brain neoplastic entities. Bar charts represent the counts of clinical symptoms for patients with underlying neoplastic ICH. Each bar indicates the number of patients (n) presenting with the listed symptom.
